# Evaluating the Probability of Survival and Damage Mechanisms of Resin Matrix Ceramics: Insights Into Wear Progression and Surface Damage Under Fatigue

**DOI:** 10.1111/jerd.70036

**Published:** 2025-09-25

**Authors:** Mirelle M. Ruggiero, Mariana I. M. Freitas, Yu Zhang, Altair A. D. B. Cury

**Affiliations:** ^1^ Department of Prosthodontics and Periodontology Piracicaba Dental School, University of Campinas Piracicaba São Paulo Brazil; ^2^ Laboratories for Microstructure Physics & Mechanics of Materials, Department of Preventive and Restorative Sciences School of Dental Medicine, University of Pennsylvania Philadelphia Pennsylvania USA

**Keywords:** ceramics, dental veneers, polymer‐infiltrated ceramic networks, stress, volume loss

## Abstract

**Objective:**

This study evaluated the probability of survival, failure modes, and fatigue‐induced wear progression, as well as assessed damage tolerance under simulated mastication in two resin matrix ceramics (polymer‐infiltrated ceramic networks (PICN) and resin nanoceramic (RNC)).

**Methodology:**

Specimens underwent step‐stress accelerated life testing (SSALT) (*n* = 21), mouth‐motion simulation (*n* = 12), followed by wear analysis and flexural strength testing. Probability of survival and flexural strength were analyzed using Weibull statistical modeling. Damage mechanisms were characterized by optical microscopy, scanning electron microscopy (SEM), and high‐resolution 3D quantitative analysis of wear depth and volumetric loss after mouth motion simulation. Two‐way ANOVA was used, followed by Tukey Post Hoc with a significance level of 5% (*α* = 0.05).

**Results:**

At 600 N, PICN exhibited significantly lower probability of survival (6%) compared to RNC (60%). SEM images showed delamination and radial cracking for both groups, being more frequent in PICN. Both materials demonstrated progressively significant increases in volumetric wear with the increase in the number of cycles and showed 0.014 mm^3^ for PICN and 0.016 mm^3^ for RNC after 100,000. Flexural strength was higher for RNC (303 MPa) than for PICN (189 MPa). Also, it decreased after 1,000 cycles for PICN, while for RNC it decreased only after 100,000 cycles, and it was always higher than for PICN. SEM images showed that fracture originated at wear craters.

**Conclusions:**

RNC exhibited superior probability of survival, particularly under more demanding conditions compared to PICN. Both materials showed a progressive increase in surface wear as the number of cycles increased. The flexural strength of RNC remained consistently higher over time. Regarding failure mechanisms, both materials experienced delamination and crack formation; however, radial cracks were more frequently observed in PICN, indicating a higher susceptibility to structural failure under fatigue conditions.

**Clinical Significance:**

Selection of restorative materials must balance mechanical performance and long‐term durability under functional loading. Resin nanoceramic appears to be a more reliable option than polymer‐infiltrated ceramic networks for restorations in high‐stress areas, such as posterior teeth or patients with parafunctional habits.

## 1 Introduction

Resin matrix ceramics (RMCs) have emerged as a promising option for restoring teeth affected by wear. RMCs exhibit reduced brittleness compared to conventional ceramics [1], providing greater resilience to functional loading and possibly making them more suitable for high‐stress clinical scenarios. RMCs combine advantageous polymer properties, such as elasticity [2] and energy absorption, with the esthetic and mechanical benefits of ceramics [3, 4, 5]. Among the RMCs, one is the resin nanoceramic (RNC), composed of approximately 80% silica and zirconia nanoparticles dispersed in a polymer matrix [6], and another is the polymer‐infiltrated ceramic network (PICN) consisting of 86% feldspathic ceramics infiltrated with a polymer [4, 5, 7]. RMCs demonstrate favorable fatigue resistance [8, 9] and may be suitable for patients with parafunctional habits [10]. However, they tend to exhibit greater susceptibility to wear compared to conventional ceramics, with studies highlighting wear as a critical factor influencing their long‐term clinical success [11].

Wear resistance is a key clinical parameter for evaluating the durability of restorative materials under masticatory forces and oral friction [12]. It is defined as the gradual loss of material resulting from mechanical interaction between two contact surfaces in relative motion under load [13]. This process involves the physical separation of material components, primarily due to microfractures and chemical dissolution, leading to characteristic wear patterns [13]. Four principal types of wear are typically identified: adhesive, abrasive, fatigue, and corrosive wear [13].

Among these, abrasive wear is the most frequently observed in restorative materials. It is primarily driven by a plowing mechanism [14], in which indenting asperities or hard particles from the opposing surface create grooves, potentially resulting in microfractures in brittle materials [13]. In contrast, fatigue wear occurs under repeated elastic contact and is characterized by crack propagation originating from surface microdefects on the indenter [13]. The cyclic nature of this loading favors crack growth, which may eventually reach the surface, causing delamination, fragmentation, and material loss in the vicinity of the indentation.

Fatigue‐related damage is particularly concerning in materials such as ceramics. During sliding contact, partial cone cracks may form at the trailing edge of a blunt indenter due to friction‐induced tensile stresses, requiring lower occlusal loads for crack initiation compared to purely axial loading [15, 16]. These mechanisms can significantly compromise the structural integrity of restorative materials in relatively short periods [17], raising concerns about how deeply penetrating partial cone cracks may reduce the functional longevity and mechanical performance of ceramics.

Studies investigating wear in CAD/CAM materials report variable wear rates and surface damage depending on testing protocols. However, limited data exist on fatigue‐related damage, which appears to play a crucial role in the long‐term success of these restorations [17]. High‐load fatigue testing on anatomical specimens provides valuable insights into ultimate strength and failure mechanisms, simulating critical occlusal forces under extreme conditions. Conversely, wear simulations under lower cyclic loads replicate the gradual degradation during routine mastication, revealing progressive material loss and surface deterioration. Together, these complementary methodologies offer a comprehensive understanding of strength degradation: high‐load fatigue tests identify catastrophic failure thresholds. At the same time, low‐load wear simulations elucidate cumulative damage relevant to long‐term clinical performance.

Therefore, this study evaluated the probability of survival, failure modes, and fatigue‐induced wear progression, as well as assessed damage tolerance under simulated mastication. Based on the current knowledge, the research hypotheses are: (1) RNC and PICN will present similar fatigue resistance due to their dual‐phase (polymer–ceramic) structure; (2) the progression of wear in these materials will negatively influence their fatigue performance and damage tolerance; (3) RNC will demonstrate greater cumulative wear progression than PICN under fatigue and simulated mastication; and (4) both RMCs will exhibit comparable failure modes.

## 2 Methodology

**FIGURE 1:**
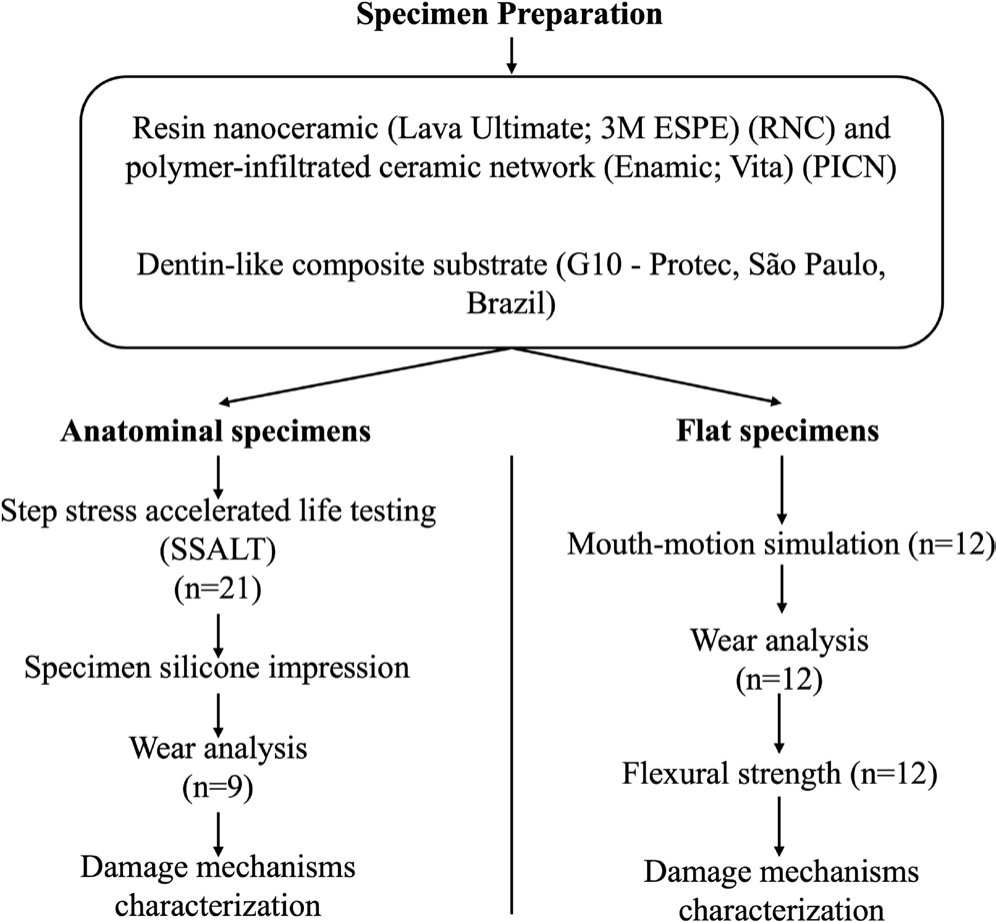
Flowchart of the experimental design.

Flowchart of the experimental design is shown in Figure 1.

### 2.1 Specimen Preparation

An occlusal veneer model of a mandibular first molar was designed using CAD software (Ceramill Mind; Amann Girrbach, Koblach, Austria) with a thickness of 1.5 mm. This model was milled in two different materials (*n* = 21): resin nanoceramic (Lava Ultimate; 3 M ESPE, St. Paul, USA) and polymer‐infiltrated ceramic network (Enamic; Vita, Bad Sackingen, Germany), resulting in a total of 42 occlusal veneers, with 21 fabricated from each material using a milling machine (Ceramill Motion 2—Amann Girrbach). The sample size was determined based on prior studies that defined the minimum number of specimens necessary to ensure robust and reliable statistical analysis for this methodological approach [18]. Finishing and polishing were performed according to the manufacturers' recommendations, using the abrasive rubber kit specified by each manufacturer.

In the same milling machine, 21 corresponding dentin‐like composite substrates were fabricated by reducing the CAD model of the mandibular first molar and reproducing it using an epoxy resin composite material (G10—Protec, São Paulo, Brazil) for each study group.

The dentin‐like composite substrates were embedded in self‐curing acrylic resin and, before cementation, were aged in distilled and deionized water at 37°C for 21 days to allow complete hydration [19]. Dentin‐like composite substrates were etched with 10% hydrofluoric acid (Condac porcelana; FGM Produtos Odontológicos) for 120 s. The occlusal veneers pretreatment followed manufacturer protocols: RNC was sandblasted with 50 μm aluminum oxide (Óxido de Alumínio, Bio‐Art) at 2 bar pressure for 5 s at ~1 cm distance, while PICN was etched with 5% hydrofluoric (Condac porcelana; FGM Produtos Odontológicos) acid for 60 s. Both substrates and occlusal veneers were then ultrasonically cleaned in distilled water for 2 min. A universal adhesive (Single Bond Universal; 3 M Oral Care) was applied for 20 s to both the ceramic and substrate, followed by dual‐cure resin cement application (RelyX Ultimate; 3 M Oral Care) to the internal surface of the occlusal veneer. The restoration was seated under a constant 10 N load to ensure uniform cement distribution. Excess cement was removed with a micro‐brush, and margins were light‐cured (radii‐cal; SDI Limited Bayswater) for 20 s per surface [11]. After cementation, all specimens were stored in distilled water at 37°C for 5 days before undergoing step‐stress accelerated life testing.

Additionally, 12 flat specimens (*n* = 12) [20] per group were prepared from CAD‐CAM blocks composed of the same material as the anatomical (occlusal veneer) specimens. These blocks were first shaped into rods 12 mm in diameter and then were sectioned into discs using a diamond blade on a low‐speed cutting machine with water cooling (Isomet Low Speed Saw, Buehler, Lake Bluff, IL, USA). The resulting discs were then sequentially polished using silicon‐carbide abrasive papers (MicroCut S Discs) with progressively finer grit sizes (#320, #800, #1200, #1400; Buehler, Lake Bluff, IL, USA) until achieving final dimensions of 1.2 mm in thickness and 12.0 mm in diameter.

The RMC discs were cemented onto a dentin‐like composite substrate (G10—Protec, São Paulo, Brazil) following the same cementation protocol used for the anatomical specimens, ensuring consistency in the bonding process. The specimens were kept in distilled water at 37°C for 5 days before the mouth‐motion simulation.

### 2.2 Step Stress Accelerated Life Testing (SSALT)

Three specimens from each anatomical occlusal veneer (OV) group were subjected to the single load‐to‐failure (SLF) test using a universal testing machine (Instron 4411, Corona, CA, USA) with a load applied axially through a tungsten carbide indenter on the central fossa of the occlusal surface of the OV using a 5 kN load cell at a loading rate of 1 mm/min. Based on the SLF data, three different step‐stress profiles, mild, moderate, and aggressive, were designed [18]. Specifically, the profiles were defined using the mean SLF value as a reference, starting at approximately 30% and ending at around 60% of that value. The terms mild, moderate, and aggressive reflect the increasingly rapid stepwise loading sequences, where specimens in the mild profile are cycled for a longer duration to reach the same final load as those in the other profiles. This approach aims to distribute failures across different load levels, enhancing the robustness of statistical modeling. The remaining specimens (*n* = 18/group) were allocated to the three fatigue profiles: mild (*n* = 9), moderate (*n* = 6), and aggressive (*n* = 3), following the distribution ratio of 3:2:1, respectively, considering that the accuracy of statistical prediction is inversely proportional to its cycling length [18]. The sample size was determined based on prior studies that defined the minimum number of specimens necessary to ensure robust and reliable statistical analysis for this methodological approach [18].

The SSALT test was performed on an electrodynamic fatigue testing machine (ElectroPlus E3000 Linear‐Torsion Test InstrumentTM; Instron, Norwood, MA, USA) in the presence of distilled water at 20 Hz [21]. In each cycle, the tungsten carbide indenter contacted 0.5 mm lingual to the distobuccal cusp tip of the occlusal veneer in an axial direction. The test was finished when samples failed (considered as chipping or delamination) or when suspended for no event being detected throughout the designed cycles and 1500 N maximum load.

Based on the SSALT failure distribution, use‐level probability Weibull curves were calculated and plotted (Synthesis 9, Alta Pro; Reliasoft, Tucson, AZ, USA) using the Weibull distribution and the inverse power law life‐stress relationship for damage accumulation. The probability of survival was calculated for completion of a mission of 100,000 cycles at 200, 300, 400, 500, and 600 N, and the differences between groups were identified based on the non‐overlap of the 90% two‐sided confidence interval (CI). This analysis provided the beta (β) value, which described the behavior of the failure rate over time (β < 1: values indicated that the failure rate had decreased over time, β ~1: failure rate did not vary over time, and β > 1: meant that the failure rate had increased over time). If the calculated beta value was < 1 for any group, then a Weibull 2‐Parameter Contour plot (Weibull modulus—m vs. characteristic strength eta—η) was computed using the final load to failure data of all groups [18].

SSALT samples were first inspected under a polarized light stereomicroscope (AxioZoom V.16, Zeiss, Oberkochen, Germany) and then observed in a scanning electron microscope (SEM) (Quanta 600 FEG ESEM) for failure mode analysis [18].

#### 2.2.1 Wear Analysis

In order to investigate the wear progression of the occlusal veneers, the SSALT cycles were interrupted [22]. For 9 specimens from each group (3 specimens from each profile) at each progression of 150 N and ended when reaching the load of 500 N. The sample size was determined based on previous studies employing a similar methodology [9]. The maximum load was defined as a value lower than the characteristic strength (corresponding to the load at which 63% of the specimens would fracture) [11] and within the range of posterior teeth bite forces [23, 24].

At each interruption, a specimen impression was made using silicone (Panasil—Ultradent, USA) without the specimen being removed from its position. Thus, the SSALT was resumed at the same position, number of cycles, and load at which it was interrupted [22].

To evaluate the volume loss across all groups, high‐resolution scanning (10 μm) of the wear craters on the silicon impression was performed using a Laser Scanner (SD Mechatronic Laser Scanner LAS‐20) [25]. Images of the samples were captured from the impression before and after the SSALT, then aligned and subtracted to isolate the wear scar and calculate the volume loss. The wear volume loss of the specimens was analyzed quantitatively with 3D reconstruction software (3D System Geomagic Wrap) [25]. The original and worn surface models were superimposed, and the two models were then subtracted to generate a third model, which was used to calculate the scar volume.

### 2.3 Mouth Motion Simulation and Damage Tolerance

The specimens underwent cyclic loading with a contact‐slide‐liftoff mouth‐motion simulation using an electrodynamic fatigue testing machine (EnduraTEC ELF 3300, TA Instrument, Minnetonka, MN, USA) [26]. A load of 50 N was applied at the center of the RMC disc via a spherical 3Y‐TZP antagonist (*r* = 3.15 mm, surface roughness Ra = 0.254 μm) at a loading rate of 1000 N/s (~2 Hz) in distilled water [20]. To facilitate off‐axis loading of the antagonist, the specimens were positioned at a 30° inclination within the fatigue testing setup. During the test, the antagonist contacted the RMC surface, applied the maximum load while sliding 1 mm down the incline, then lifted off and returned to its initial position to start a new cycle. Three different cyclic loading durations were analyzed: 1000, 10,000, and 100,000 cycles, and a new antagonist was used for every specimen [20], and 12 (*n* = 12) specimens were used per group, with the sample size determined based on previous studies employing the same methodology [20].

#### 2.3.1 Wear Analysis

The volume loss and maximum wear depth after 1,000, 10,000, and 100,000 cycles were evaluated using high‐resolution scanning (10 μm in the xy‐plane and 5 μm along the *z*‐axis) of the wear craters on the specimens with a Laser Scanner (SD Mechatronic Laser Scanner LAS‐20) [25]. The wear volume loss and wear depth were analyzed using 3D reconstruction software (3D System Geomagic Wrap) [25]. Pre‐test scanning was not required, as the original flat surface could be reconstructed post‐test using the 3D image software. This approach mirrored the methodology used for analyzing wear in the anatomical specimens.

#### 2.3.2 Flexural Strength

Following the simulations, the ceramic specimens were detached from the G10 substrate using a diamond blade on a low‐speed cutting machine under water irrigation. Any residual G10 material was manually removed from the specimen surface. To evaluate damage tolerance, the specimens underwent biaxial flexural strength testing. The discs were tested using a piston‐on‐three‐balls setup on a universal testing machine (Instron series 5566, Instron Worldwide Headquarters, Norwood, MA, USA) at a 1 mm/min crosshead speed. The test was conducted with the damaged surface of the specimen under tensile loading. Additionally, 12 discs that had not undergone fatigue testing were evaluated for each ceramic material to determine the initial flexural strength (control). The biaxial flexural strength (MPa) was calculated using the ISO/FDIS 6872:2024(E) equations.

$$\sigma =\frac{-0.2387p\left(X-Y\right)}{b^{2}}$$

where

$$X=\left(1+\upsilon \right)\ln{\left(r_{2}/r_{3}\right)}^{2}+\left[\left(1-\upsilon \right)/2\right]{\left(r_{2}/r_{3}\right)}^{2}$$

$$Y=\left(1+\upsilon \right)\left[1+\ln{\left(r_{1}/r_{3}\right)}^{2}\right]+\left(1-\upsilon \right){\left(r_{1}/r_{3}\right)}^{2}$$

where *r*_1_ = 5.00 mm (radius of support circle), *r*_2_ = 0.70 mm (radius of the loaded area), *r*_3_ = 7.00 mm (disc radius), *b* = 1.20 mm (disc thickness), *v* = 0.24 (Poisson's ratio for PICN) and *v* = 0.45 (Poisson's ratio for RNC) [27], and *P* = the maximum load the specimen can withstand before a fracture occurs (N).

### 2.4 Surface Damage Mechanisms Characterization

Specimens from each SSALT test group, previously used in volumetric wear analysis, were selected to characterize surface damage mechanisms caused by wear progression on the restoration surfaces and fractography patterns [22]. Initially, specimens were examined under transilluminated light in a stereomicroscope (AxioZoom V.16, Zeiss, Oberkochen, Germany) to highlight surface damage characteristics, such as crack systems. Further analysis was conducted using scanning electron microscopy (SEM) (Quanta 600 FEG ESEM).

The damage introduced by mouth‐motion simulation was assessed through SEM imaging (Quanta 600 FEG ESEM). Surface damage was mapped by analyzing the wear scar. After biaxial flexural strength testing, the fracture surfaces were examined using an optical microscope (Nikon LV150, Tokyo, Japan) to identify fractographic features related to crack propagation and after that were analyzed in SEM (Quanta 600 FEG ESEM).

### 2.5 Statistical Analysis

Statistical data analysis was performed using GraphPad Prism 9 (GraphPad Software, San Diego, CA, EUA) statistics software. The two‐way ANOVA (factors: material and load/number of cycles) was used to analyze volume loss, maximum wear depth, and flexural strength data of PICN versus RNC, and the Tukey Post Hoc test was used to identify significant differences between groups, with a significance level of 5% (*α* = 0.05).

## 3 Results

### 3.1 Step Stress Accelerated Life Testing (SSALT)

**FIGURE 2:**
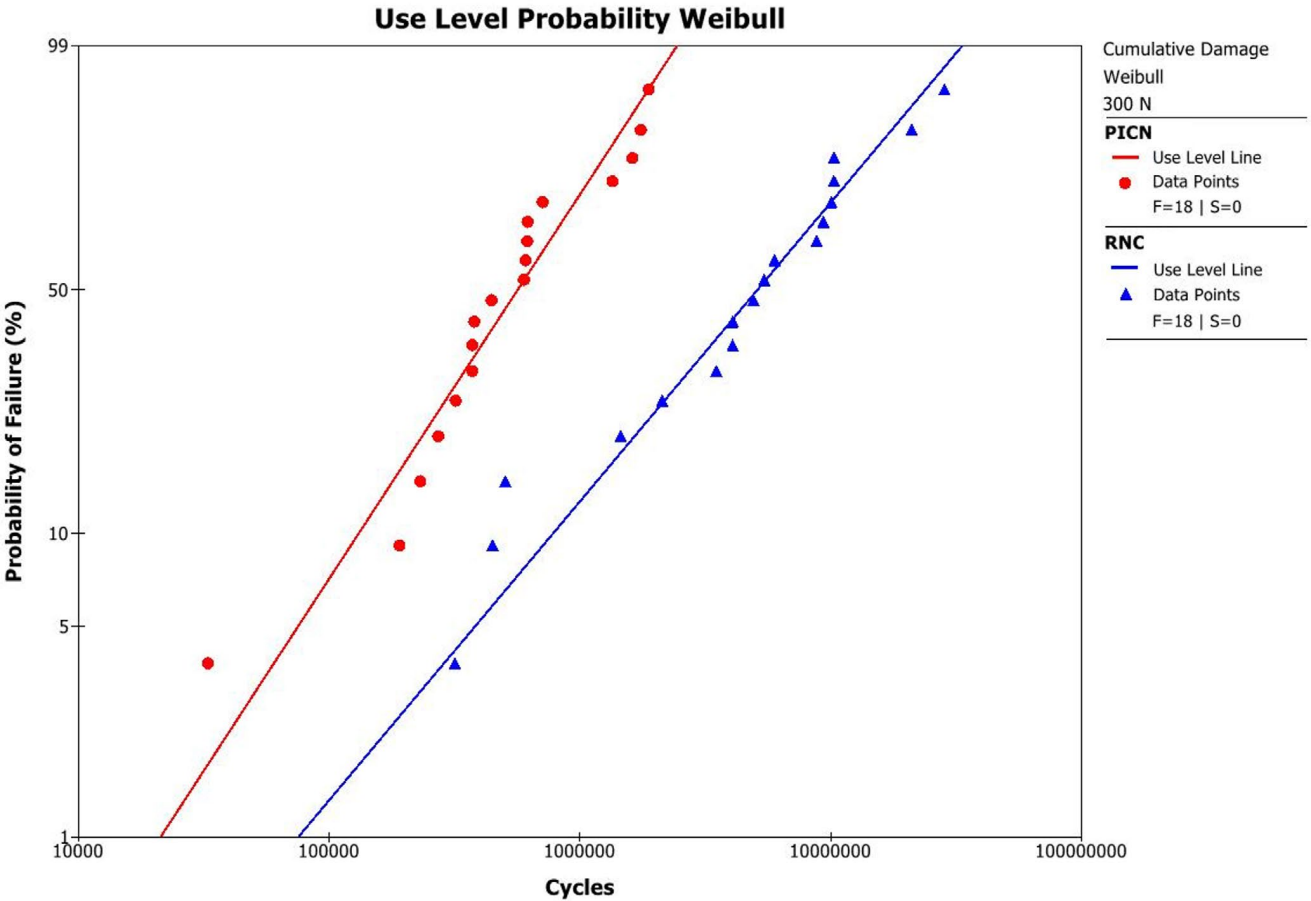
Use‐level probability Weibull plot showing the probability of failure as a function of cycles for the PICN and RNC occlusal veneers.

Use‐level probability Weibull curves for occlusal veneers under a 300 N load are shown in Figure 2. The mean beta (β) values for PICN and RNC were greater than 1, indicating an increasing failure rate over time due to fatigue damage accumulation (Table 1).

**TABLE 1:** Calculated probability of survival (%) for a given mission of 100,000 cycles at set loads of 200, 300, 400, 500, and 600 N for materials.

		200 N	300 N	400 N	500 N	600 N	Beta (β)
	Upper bound	100	98	86	56	22	3.26
PICN	Probability of survival (%)	99 Aa	93 Aab	72 Abc	35 Acd	6 Ad	1.29
	Lower bound	95	80	49	15	0	0.51
	Upper bound	100	100	99	94	79	2.51
RNC	Probability of survival (%)	100 Aa	99 Aab	94 Aabc	82 Abc	60 Bc	1
	Lower bound	98	91	77	56	32	0.4

*Note*: Different uppercase letters mean statistical difference between materials. Different lowercase letters represent statistical difference between missions. Differences between groups were identified based on the non‐overlap of 90% two‐sided confidence interval.

Table 1 presents the probability of survival for a mission of 100,000 cycles under loads ranging from 200 to 600 N. At 600 N, PICN exhibited a significantly lower probability of survival compared to RNC. At 200 and 300 N, both materials showed survival probabilities above 90%. However, the PICN probability of survival decreased significantly between 200 and 400 N, and again from 400 to 600 N. In contrast, RNC showed a significant reduction in probability of survival at 500 N compared to 200 N.

**FIGURE 3:**
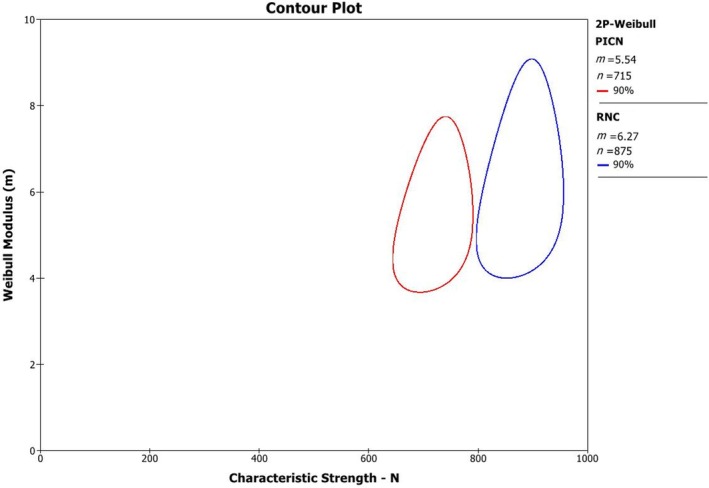
Contour plot showing Weibull modulus (m) and characteristic strength (η).

The 2‐parameter Weibull contour plot (Weibull modulus vs. characteristic strength) is shown in Figure 3. The Weibull modulus ranged from 5.54 to 6.27 with no significant difference between groups. However, RNC exhibited a significantly higher characteristic strength (875 N) than PICN (715 N) (Table 2).

**TABLE 2:** Characteristic Strength and Weibull modulus for both materials.

	PICN	RNC
Upper bound	770	933
Characteristic strength	715 A	875 B
Lower bound	664	819
Upper bound	7.34	8.56
Weibull modulus	5.54 A	6.27 A
Lower bound	4.18	4.6

*Note*: Different uppercase letters represent statistical differences between materials. Differences between groups were identified based on the non‐overlap of 90% two‐sided confidence interval.

#### 3.1.1 Wear Analysis

Table 3 presents the volumetric wear loss (mm^3^) observed for PICN and RNC materials under two load conditions (300 and 500 N). The volumetric wear loss of both materials, PICN and RNC, was significantly affected by both load (*p* < 0.0001) and material (*p* = 0.0493), and there was interaction between the two variables (*p* = 0.0063). At 300 N, PICN exhibited significantly lower wear loss (0.039 ± 0.010 mm^3^) compared to RNC (0.077 ± 0.045 mm^3^) (*p* = 0.0122). However, under the 500 N load, no statistically significant difference was observed between PICN (0.280 ± 0.091 mm^3^) and RNC (0.233 ± 0.020 mm^3^) (*p* = 0.7751). When comparing the load effects within each material, both PICN and RNC showed significantly greater wear at 500 N compared with 300 N.

**TABLE 3:** Volumetric wear loss (mm^3^) for both materials.

	PICN	RNC
300 N	0.039 ± 0.010 Aa	0.077 ± 0.045 Ba
500 N	0.280 ± 0.091 Ab	0.233 ± 0.020 Ab

*Note*: Different uppercase letters represent statistical difference between materials. Different lowercase letters represent statistical difference between cycles. Differences between groups were identified by two‐way ANOVA followed by Tukey's post hoc and significance level *α* = 0.05.

### 3.2 Mouth Motion Simulation and Damage Tolerance

The volumetric wear loss of PICN and RNC materials after 1000, 10,000, and 100,000 wear cycles is presented in Table 4. The volumetric wear loss of both materials, PICN and RNC, was significantly affected by the number of cycles (*p* < 0.0001), but not by the material (*p* = 0.1319), and there was no interaction between the two variables (*p* = 0.2100). Both materials exhibited a statistically significant increase in wear loss between 1000 and 10,000 cycles (*p* < 0.05), while no significant differences were observed between 10,000 and 100,000 cycles for either material. After 1000 cycles, PICN and RNC showed similar volumetric wear loss (0.005 ± 0.002 mm^3^ and 0.003 ± 0.000 mm^3^, respectively), with no statistically significant difference between the materials. This trend persisted at 10,000 cycles and 100,000 cycles, and no statistical difference between groups was observed.

**TABLE 4:** Volumetric wear loss (mm^3^) for both materials.

Cycles	PICN	RNC
1000	0.005 ± 0.002 Aa	0.003 ± 0.000 Aa
10,000	0.013 ± 0.000 Ab	0.012 ± 0.003 Ab
100,000	0.014 ± 0.003 Ab	0.016 ± 0.002 Ab

*Note*: Different uppercase letters represent statistical differences between materials. Different lowercase letters represent statistical difference between cycles. Differences between groups were identified by two‐way ANOVA followed by Tukey's post hoc and significance level *α* = 0.05.

The depth of the wear crater for both PICN and RNC materials after 1000, 10,000, and 100,000 cycles is presented in Table 5. The depth of the wear crater of both materials, PICN and RNC, was significantly affected by both cycles (*p* < 0.0001) and material (*p* = 0.0026) and there was interaction between the two variables (*p* = 0.0006). At 1000 cycles, both materials exhibited similar crater depths (0.041 ± 0.005 mm and 0.033 ± 0.002 mm, respectively), with no statistically significant difference between groups. For PICN, no significant difference in crater depth was observed between 1000 and 10,000 cycles. However, a significant increase was noted at 100000 cycles. In contrast, RNC exhibited a progressive and statistically significant increase in crater depth across all cycles: 0.033 ± 0.002 mm (1000 cycles), 0.047 ± 0.008 mm (10,000 cycles), and 0.059 ± 0.005 mm (100,000 cycles). Furthermore, RNC demonstrated significantly greater crater depths than PICN at both 10,000 and 100,000 cycles.

**TABLE 5:** Depth of wear crater (mm) for both materials.

Cycles	PICN	RNC
1000	0.041 ± 0.005 Aa	0.033 ± 0.001 Aa
10,000	0.041 ± 0.004 Aa	0.047 ± 0.008 Bb
100,000	0.050 ± 0.005 Ab	0.059 ± 0.005 Bc

*Note*: Different uppercase letters represent statistical differences between materials. Different lowercase letters represent statistical difference between cycles. Differences between groups were identified by two‐way ANOVA followed by Tukey's post hoc and significance level *α* = 0.05.

The flexural strength of both materials, PICN and RNC, was significantly affected by both number of cycles and material (Table 6) (*p* < 0.0001) and there was interaction between the two variables (*p* = 0.0020). RNC exhibited consistently higher flexural strength than PICN across all cycling conditions (*p* < 0.05). In the control group, RNC demonstrated a flexural strength of 303 ± 11 MPa, which was significantly greater than that of PICN (189 ± 7 MPa).

**TABLE 6:** Flexural strength for both materials before and after cycling.

	PICN	RNC
Control	189 ± 7 Aa	303 ± 11 Ba
1000	121 ± 38 Ab	290 ± 26 Ba
10,000	101 ± 10 Ab	269 ± 26 Ba
100,000	94 ± 5 Ab	207 ± 22 Bb

*Note*: Different uppercase letters represent statistical difference between materials. Different lowercase letters represent statistical difference between cycles. Differences between groups were identified by two‐way ANOVA followed by Tukey's post hoc and significance level *α* = 0.05.

Following 1000 cycles, PICN experienced a marked reduction in flexural strength (121 ± 38 MPa), whereas RNC maintained values comparable to its control condition (290 ± 26 MPa). As the number of cycles increased to 10,000 and 100,000, PICN continued to show decreasing values—101 ± 10 and 94 ± 5 MPa, respectively—with no statistically significant difference between these two groups. In contrast, RNC showed a progressive decline, reaching 269 ± 26 MPa at 10,000 cycles and 207 ± 22 MPa at 100,000 cycles, with statistical significance observed only at 100,000.

### 3.3 Surface and Subsurface Damage Mechanisms Characterization

**FIGURE 4:**
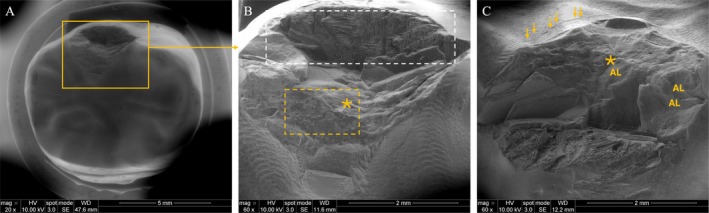
SEM micrographs of a representative delaminated surface of a PICN specimen, showing occlusal (A and B) and buccal (C) views of the failure. Images (A) and (B) correspond to low and high magnifications, respectively, of the occlusal surface. In (B), wear is visible as dark gray regions (highlighted by a yellow dotted square), resulting from contact with the indenter during fatigue loading. The asterisk (*) indicates the fracture origin in the loading area, where the underlying epoxy resin is exposed (white dotted square). Image (C) provides a buccal view, displaying arrest lines (AL) with their concave orientation pointing toward the fracture origin (*), and radial fracture patterns indicated by arrows.

**FIGURE 5:**
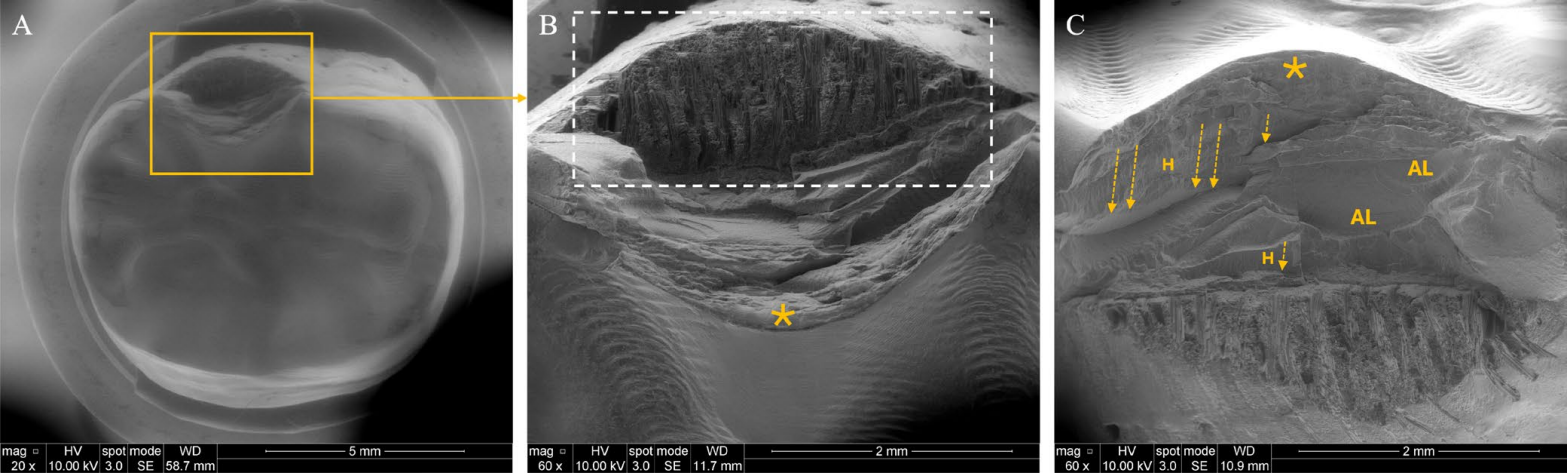
SEM micrographs of an RNC specimen's representative delaminated surface illustrate occlusal (A and B) and buccal (C) views of the failure. Images (A) and (B) correspond to low and high magnifications of the occlusal surface, respectively. In B, the asterisk (*) marks the fracture origin in the loading area, where the underlying epoxy resin is exposed (white dotted square). Image (C) shows the direction of crack propagation, indicated by dotted arrows and supported by multiple hackle lines (H). Also, arrest lines (AL) are observed, with concave portions oriented toward the fracture origin (*).

The SSALT samples predominantly failed by delamination (Figures 4 and 5), evidenced by adhesive detachment exposing the underlying epoxy resin. Prior to fracture, occlusal surface wear due to contact with the indenter was consistently detected. A competing failure mechanism was identified between the occlusal surface—specifically beneath the load application site, where hackle and arrest lines, indicating its point of origin, were present—and the intaglio surface, which displayed radial cracks for both groups. However, radial cracks were more frequently observed in PICN (Figure 4).

**FIGURE 6:**
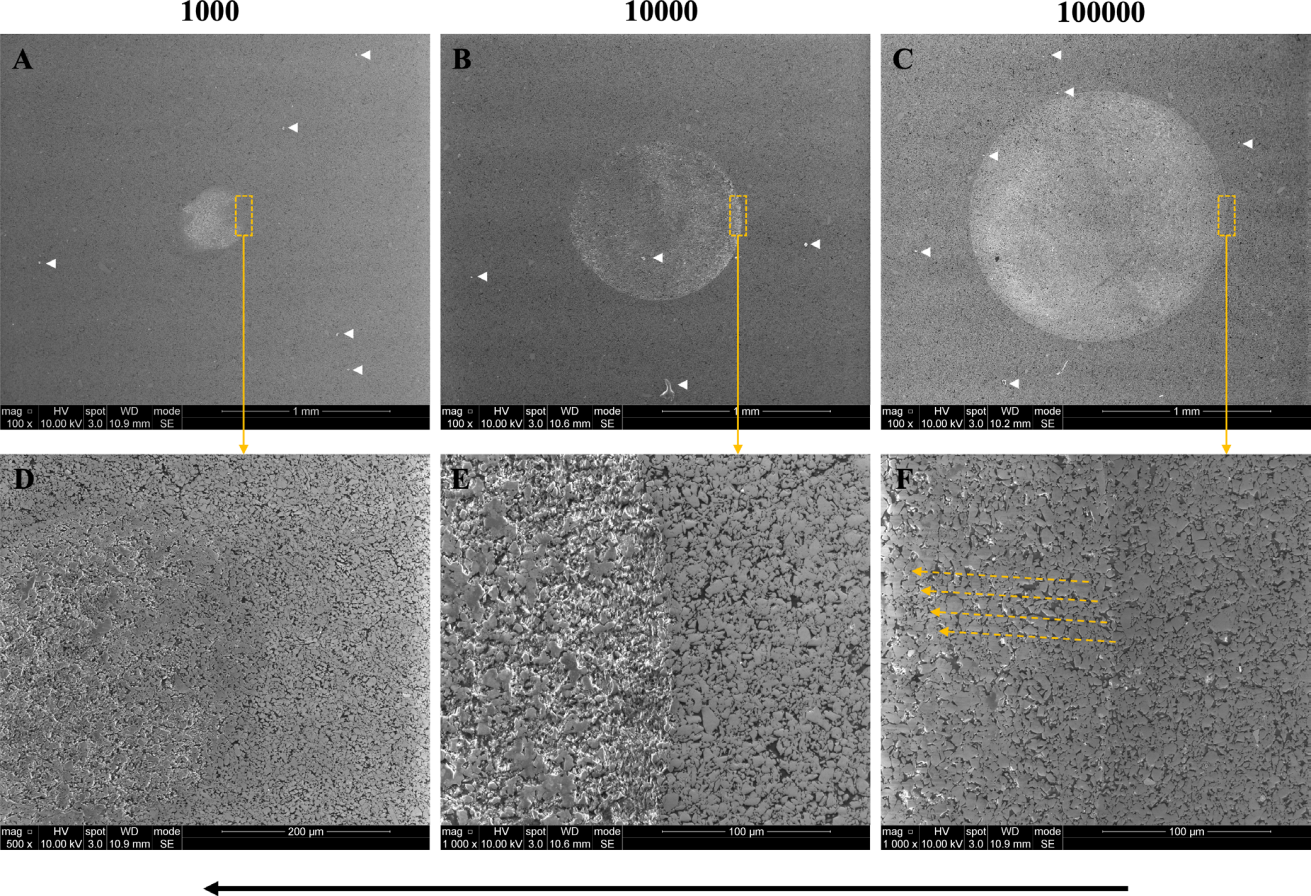
Representative images of PICN surfaces after cyclic loading of 50 N for 1000 (A), 10,000 (B), and 100,000 (C) cycles. A progressive increase in the wear crater area is observed as the number of cycles increases. Images (D–F) show higher magnifications of (A–C), respectively, as indicated by the yellow dotted squares and arrows. The black arrow indicates the direction of the indenter during the mouth‐motion simulation. White arrows highlight debris fragments, while yellow dotted arrows indicate the direction of the scratches formed by abrasion from these fragments.

**FIGURE 7:**
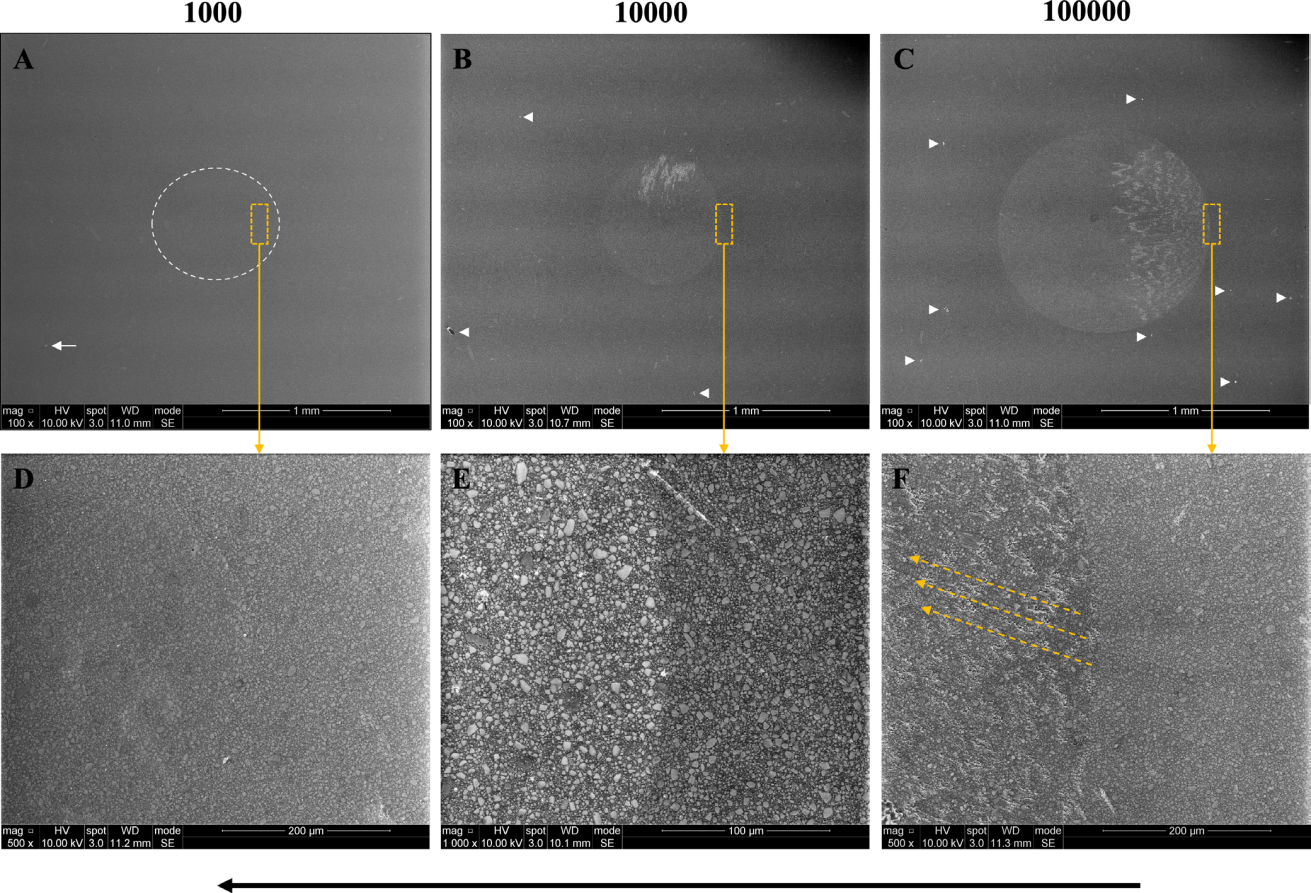
Representative images of RNC surfaces after cyclic loading of 50 N for 1000 (A), 10,000 (B), and 100,000 (C) cycles. A progressive increase in the wear crater area is observed as the number of cycles increases. Images (D–F) show higher magnifications of (A–C), respectively, as indicated by the yellow dotted squares and arrows. The black arrow indicates the direction of the indenter during the mouth‐motion simulation. The white dotted circle surrounds the almost imperceptible wear crater after 1000 cycles. White arrows highlight debris fragments, while yellow dotted arrows indicate the direction of the scratches formed by abrasion from these fragments.

For the mouth‐motion simulation specimens, representative images of the surfaces of PICN and RNC after cyclic loading of 50 N for 1,000, 10,000, and 100,000 cycles are shown in Figures 6 and 7, respectively. At the beginning of the mouth‐motion simulation, a minor oval‐shaped depression was observed along the path of the sliding indenter in both materials (Figures 6A and 7A). As the number of cycles increased, wear craters began to form (Figures 6B,C and 7B,C). Within these craters, loss and/or detachment of the polymer matrix was observed in both materials, as evidenced by the exposure of the ceramic network in PICN (Figures 6E,F) and of inorganic particles in RNC (Figure 7E,F), highlighted by the presence of a whitish halo surrounding these structures. Scattered debris fragments were visible on the specimen surface (Figures 6 and 7), likely resulting from particle detachment caused by sliding contact between the indenter and the specimen surface. It is hypothesized that these particles were initially located within the crater and acted as third bodies between the indenter and the specimen, contributing to increased surface wear. However, prior to SEM analysis, the specimens underwent ultrasonic cleaning, which may have caused these particles to disperse across the surface. Additionally, the wear crater appeared to be slightly larger in PICN (Figure 6) compared to RNC (Figure 7). Moreover, throughout the cycles, it is possible to observe that these dispersed fragments gradually created marks, grooves, or scratches on the worn surface of the specimens. The yellow dotted arrows indicate the direction of these scratches.

**FIGURE 8:**
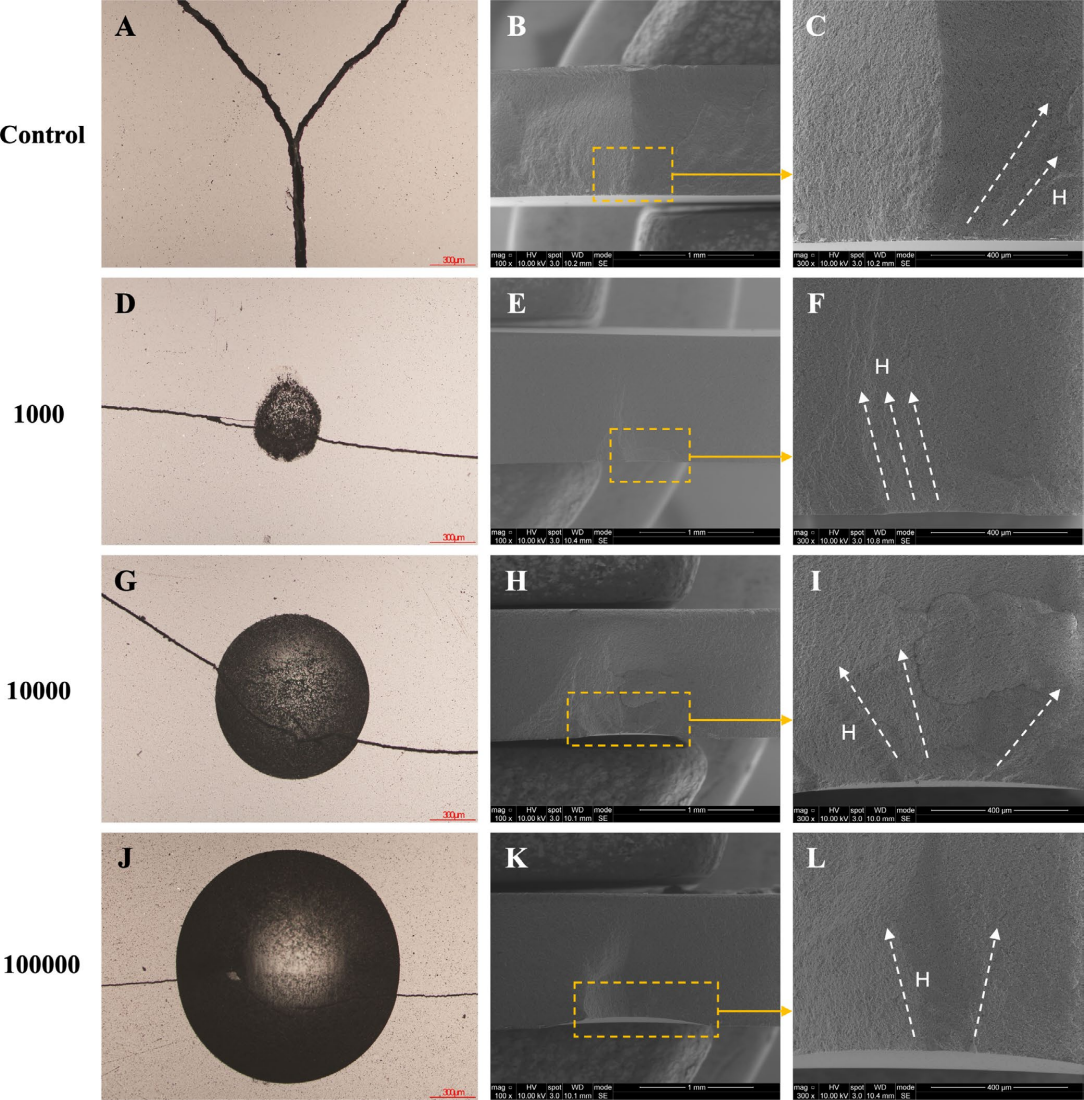
Fractographic analysis of specimens from both the PICN control group and those subjected to mouth‐motion simulation. Images (A), (D), (G), and (J) were obtained using an optical microscope, while images (C), (F), (I), and (L) show higher‐magnification SEM views corresponding to (B), (E), (H), and (K), respectively. Yellow dotted squares and arrows indicate the origin of failure—note the wear craters in (E), (H), and (K). White dotted arrows show the direction of crack propagation, as evidenced by the hackle lines (H).

Figure 8 presents the fractographic analysis of specimens from both the PICN control group and those subjected to the mouth‐motion simulation. In the control group, failure originated from natural flaws inherent to the material. In contrast, specimens exposed to mouth‐motion simulation exhibited failures initiating at the wear crater or indentation sites. All fractures involved partial cone cracks, with crack initiation located within the original cone, as indicated by the presence of hackle lines.

**FIGURE 9:**
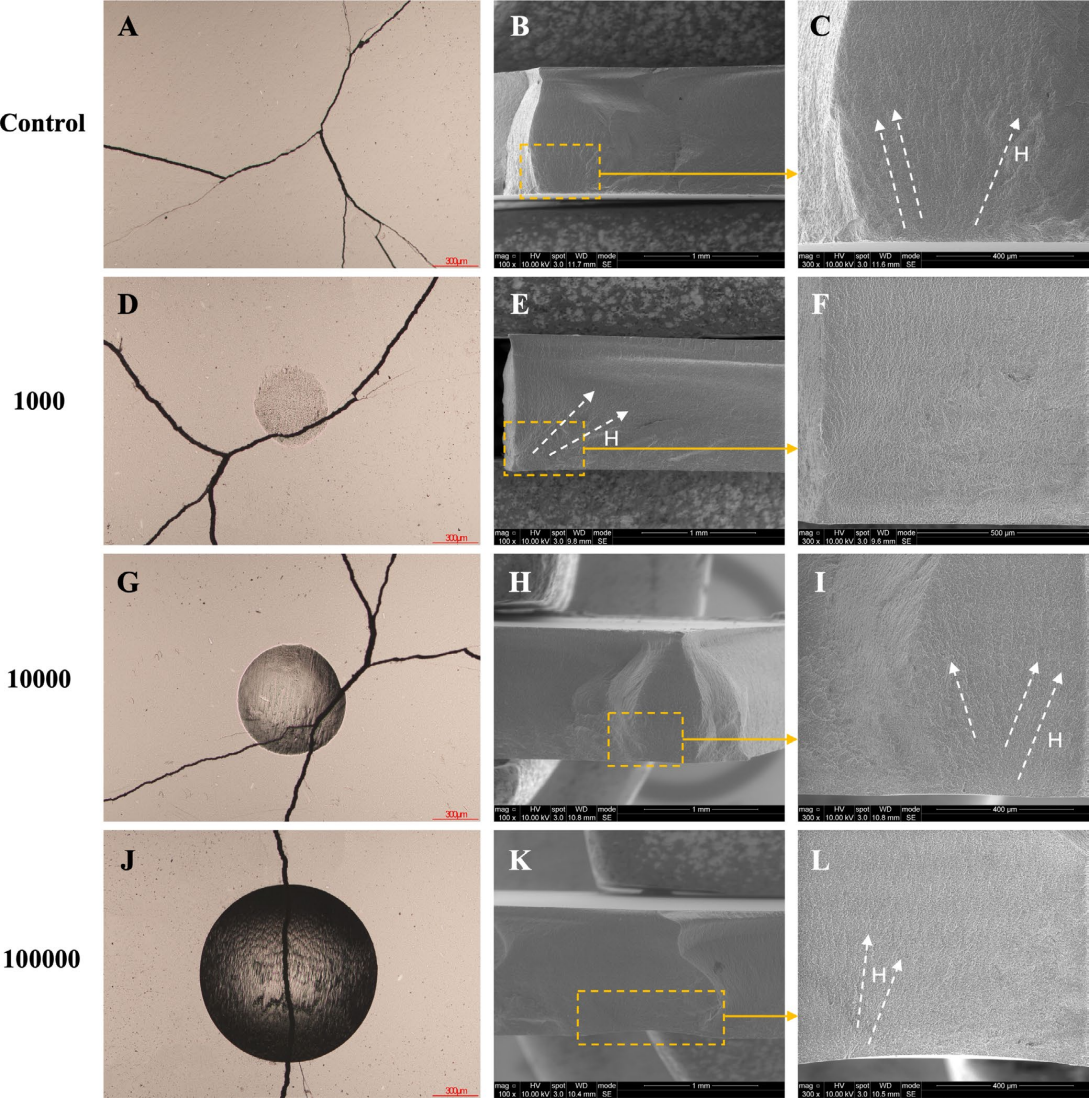
Fractographic analysis of specimens from both the RNC control group and those subjected to mouth‐motion simulation. Images (A), (D), (G), and (J) were obtained using an optical microscope, while images (C), (F), (I), and (L) show higher‐magnification SEM views corresponding to (B), (E), (H), and (K), respectively. Yellow dotted squares and arrows indicate the origin of failure—note the wear craters in (H), and (K). White dotted arrows show the direction of crack propagation, as evidenced by the hackle lines (H).

Figure 9 presents the fractographic analysis of specimens from both the RNC control group and those subjected to mouth‐motion simulation. In the control and 1000 groups, failure originated from natural flaws inherent to the material. In contrast, specimens from the 10,000 and 100,000 groups exhibited crack propagation initiating at the wear crater, as indicated by the presence of hackle lines.

## 4 Discussion

This study contributes to clinical applications, where the choice of restorative materials must balance mechanical performance and long‐term durability under functional loading. Four hypotheses were tested. The first stated that RNC and PICN would present similar fatigue resistance due to their dual‐phase microstructure. This was partially confirmed: both performed similarly under 200 and 300 N, but RNC showed higher survival at 600 N. The second predicted that wear progression would negatively affect fatigue performance and damage tolerance. Results supported this, as wear reduced fatigue strength, although RNC was less affected than PICN. The third proposed that RNC would show greater cumulative wear than PICN. This was confirmed at 300 N in the fatigue test, but no differences were observed under simulated mastication at 50 N. Finally, the fourth suggested comparable failure modes. Both materials predominantly showed delamination under fatigue, confirming similar failure patterns. In damage tolerance tests, most specimens failed at the wear crater.

The SSALT data demonstrated high survival rates—up to 100%—at load levels of 200 and 300 N for a mission of 100,000 cycles, with no statistically significant differences observed between the materials. Although the maximum bite force in the first molar region of adults without a history of parafunctional habits varies considerably across studies, the range of 200 to 500 N is widely accepted in the literature for this region [23, 24]. Under high load conditions (600 N), RNC exhibited a greater probability of survival compared to PICN, which may be attributed to the PICN microstructure—characterized by the presence of microcracks along the network boundaries [5]—potentially accelerating failure mechanisms [17]. These microcracks are likely induced by stresses generated during polymerization shrinkage of the resin and the feldspathic ceramic matrix, leading to debonding between the two phases [7]. Additionally, micro‐delamination of PICN was evident in the reconstructed image scans used for volumetric wear analysis during SSALT, further explaining its lower probability of survival due to a less stable performance over time. The higher inorganic content of PICN may also contribute to its increased friability. While both materials showed similar Weibull moduli—indicating comparable homogeneity in strength distribution and overall structural consistency—the lower characteristic strength of PICN (defined as the load at which 63.2% of specimens are expected to fail) points to a greater vulnerability to failure under repetitive and intense mechanical loading.

In the anatomical crown wear test conducted during SSALT, PICN exhibited lower volumetric loss under a 300 N load. However, when the load was increased to 500 N, wear increased for both materials, resulting in comparable volumetric losses. It is important to clarify that, in addition to the progressive increase in load, there was also an increase in the number of cycles between each measurement. These findings are consistent with mouth‐motion simulation tests performed on flat specimens under lower loads (50 N), demonstrating that wear increased proportionally with the number of cycles.

In mouth motion simulation tests, both materials exhibited a significant increase in volumetric wear between 1000 and 10,000 cycles, which stabilized between 10,000 and 100,000 cycles. This behavior was expected, as similar findings have been reported by previous studies, which observed an initial phase of accelerated material degradation followed by a plateau, likely due to the early removal of superficial, less wear‐resistant components (polymer matrix) and subsequent exposure of inorganic/ceramic components [17]. However, no differences between the materials were observed. In contrast, the SSALT revealed a distinct wear pattern, where PICN exhibited less wear than RNC in the early cycles; however, as the number of cycles increased, wear progressed in both materials, ultimately showing no significant differences between them. This discrepancy between analyses can be attributed to the more extreme and clinically relevant conditions simulated by SSALT [18], including higher loads and anatomical geometry, which collectively impose more complex and severe mechanical stresses. Despite these methodological differences, both testing protocols provide valuable insights: the mouth motion simulation highlights short‐term wear performance, whereas SSALT allows evaluation of long‐term mechanical resilience and fatigue behavior under more extreme conditions.

It is important to emphasize that the SSALT protocol is not primarily designed to evaluate wear rate, but rather to predict the probability of survival of materials [18] under simulated clinical fatigue conditions. Unlike controlled wear analysis that aims to quantify gradual surface loss over time, SSALT involves progressively increasing cyclic loading, which induces cumulative damage, combining both mechanical fatigue and material degradation. As a result, a significant amount of material loss can occur during testing—not solely from surface wear, but also from subsurface crack propagation, microstructural delamination, and catastrophic failure mechanisms. This complex interaction of failure modes reflects the clinical conditions that accelerate the aging process, making SSALT a powerful tool for assessing long‐term structural performance rather than precise wear kinetics.

Flexural strength remained consistently higher in RNC, even after fatigue cycling, reinforcing its enhanced mechanical stability and resistance to fatigue‐induced degradation. These findings are in accordance with previous studies where higher flexural strength was found for RNC than PICN [2]. In contrast, PICN exhibited significant strength reductions after only 1,000 cycles, with persistently low values in subsequent cycles. These findings indicate that PICN undergoes early mechanical deterioration under repeated loading, potentially compromising its performance in restorations exposed to high occlusal forces. This trend aligns with the outcomes observed in the SSALT protocol, where RNC demonstrated a greater probability of survival and resistance to failure under progressively increasing loads. The superior fatigue performance of RNC may be attributed to its discrete filler particles dispersed within the resin matrix, making it less brittle [1] and more effective at distributing stress compared to PICN, which consists of a rigid ceramic scaffold [11]. Additionally, RNC exhibits reduced susceptibility to interfacial debonding and microcrack propagation—issues that are more commonly observed in PICN [5].

Scanning electron microscopy analysis of SSALT specimens revealed similar failure mechanisms in both materials, characterized by delamination and radial cracks originating from the bonding interface. RNC, however, demonstrated a more progressive and controlled crack pattern, indicative of greater damage tolerance. The presence of loose debris and larger craters in PICN (Figure 6), observed during the mouth motion simulation test, suggests increased structural fragility—likely due to lower cohesion between the ceramic and polymer phases. In both materials, crack propagation was initiated at the wear crater, as evidenced by the presence of hackle lines. Notably, this pattern was observed in PICN at all cycle intervals, whereas in RNC it was only present at 10,000 and 100,000 cycles, indicating a higher susceptibility to wear in PICN.

These in vitro data suggest that while PICN offers advantages in terms of initial wear resistance and surface properties, especially under moderate loads, however, its lower fatigue resistance may limit its use in posterior restorations subject to high masticatory forces. In contrast, RNC demonstrated superior structural integrity and probability of survival, making it a more suitable option for restorations in molar regions or patients with parafunctional habits. However, since both materials undergo considerable wear due to occlusal forces, the use of occlusal splints is highly recommended [10] to reduce the risk of delamination, fractures, and wear, thereby increasing the longevity of the restorations. These insights can guide clinicians in choosing materials based on specific clinical scenarios and expected functional demands.

The limitations of this study include the inherent constraints of in vitro testing, such as the inability to fully replicate the complex and variable conditions of the oral environment, including thermal cycling and patient‐specific occlusal patterns. Despite these limitations, the combination of accelerated life testing and wear simulation provides a comprehensive assessment of mechanical performance and offers valuable predictive insights. Future studies incorporating thermal and chemical aging and clinical trials are essential to confirm these findings and refine material selection protocols.

## 5 Conclusions

Within the limitations of this current study, it was concluded that:

- Research Hypothesis #1: Resin nanoceramic exhibited a superior probability of survival, particularly under more demanding conditions compared to polymer‐infiltrated ceramic network.
- Research Hypothesis #2: Progressive wear influenced fatigue performance and damage tolerance, as both materials showed a progressive increase in surface wear with the number of cycles. However, resin nanoceramic was less affected than polymer infiltrated ceramic network, since its flexural strength was consistently higher and more stable over time, while polymer infiltrated ceramic network exhibited an earlier and more pronounced decline.
- Research Hypothesis #3: Resin nanoceramic demonstrated greater cumulative wear progression than polymer‐infiltrated ceramic network under fatigue but not under simulated mastication.
- Research Hypothesis #4: Both resin matrix ceramics exhibited comparable failure modes, including delamination and crack formation, although radial cracks occurred more frequently in polymer‐infiltrated ceramic network.

## Conflicts of Interest

The authors declare no conflicts of interest.

## Data Availability

The data that support the findings of this study are available from the corresponding author upon reasonable request.
